# Biocomposites Based on Electrospun Fibers of Poly(3-hydroxybutyrate) and Nanoplatelets of Graphene Oxide: Thermal Characteristics and Segmental Dynamics at Hydrothermal and Ozonation Impact

**DOI:** 10.3390/polym15204171

**Published:** 2023-10-20

**Authors:** Svetlana G. Karpova, Anatoly A. Olkhov, Ivetta A. Varyan, Natalia G. Shilkina, Alexander A. Berlin, Anatoly A. Popov, Alexey L. Iordanskii

**Affiliations:** 1 Department of Biological and Chemical Physics of Polymers, Emanuel Institute of Biochemical Physics, Russian Academy of Sciences, 4 Kosygina Street, 119334 Moscow, Russia; karpova@sky.chph.ras.ru (S.G.K.); aolkhov72@yandex.ru (A.A.O.); anatoly.popov@mail.ru (A.A.P.); 2 Academic Department of Innovational Materials and Technologies Chemistry, Plekhanov Russian University of Economics, 36 Stremyanny Lane, 117997 Moscow, Russia; 3 N. N. Semenov Federal Research Center for Chemical Physics Academy of Science, 119991 Moscow, Russia; tashi05@list.ru (N.G.S.); berlin@chph.ras.ru (A.A.B.)

**Keywords:** nanocomposites, graphene oxide, poly(3-hydroxybutyrate), electron spin resonance, stable radical, TEMPO

## Abstract

In order to create new biodegradable nanocomposites for biomedicine, packaging, and environmentally effective adsorbents, ultra-thin composite fibers consisting of poly(3-hydroxybutyrate) (PHB) and graphene oxide (GO) were obtained by electrospinning. Comprehensive studies of ultrathin fibers combining thermal characteristics, dynamic electron paramagnetic resonance (ESR) probe measurements, and scanning electron microscopy (SEM) were carried out. It is shown that at the addition of 0.05, 0.1, 0.3, and 1% OG, the morphology and geometry of the fibers and their thermal and dynamic characteristics depend on the composite content. The features of the crystalline and amorphous structure of the PHB fibers were investigated by the ESR and DSC methods. For all compositions of PHB/GO, a nonlinear dependence of the correlation time of molecular mobility TEMPO probe (τ) and enthalpy of biopolyether melting (Δ*H*) is observed. The influence of external factors on the structural-dynamic properties of the composite fiber, such as hydrothermal exposure of samples in aqueous medium at 70 °C and ozonolysis, leads to extreme dependencies of τ and Δ*H*, which reflect two processes affecting the structure in opposite ways. The plasticizing effect of water leads to thermal destruction of the orientation of the pass-through chains in the amorphous regions of PHB and a subsequent decrease in the crystalline phase, and the aggregation of GO nanoplates into associates, reducing the number of GO-macromolecule contacts, thus increasing segmental mobility, as confirmed by decreasing τ values. The obtained PHB/GO fibrillar composites should find application in the future for the creation of new therapeutic and packaging systems with improved biocompatibility and high-barrier properties.

## 1. Introduction

The development of novel fibrillar materials with enhanced functionality through the combination of biodegradable biopolymers and their modifiers is among the challenges of modern polymer science and technology [1,2,3]. Along with the generalized groups of polylactides (PLAs) [4] and polysaccharides [5], polyhydroxyalkanoates (PHAs) [6,7,8] form one of the most numerous families of biodegradable bio-based polymers that have become widespread as a result of their environmental and biological compatibility, and due to the almost inexhaustible sources of their synthesis. The main representative of the PHA family, as well as one of the most widely used biopolymers, is a high-crystalline thermoplastic poly(3-hydroxybutyrate) (PHB) [9,10]. This polyester is close to polypropylene in a number of physicochemical characteristics [11]. It has satisfactory thermal, mechanical, and barrier properties, but these characteristics require significant improvement in the situation when innovative platforms, implants, and devices for the implementation in the diverse areas of human activity based on PHB are designed [12,13,14].

There are several ways to improve the operational parameters of PHB, among which the most effective are changing the chemical structure of polymer molecules through (bio)synthesis of its copolymers [15], mixing macromolecules to obtain new blends and composites [16,17] and introducing modifying agents [18,19,20], producing crucial effects on thermal, diffusional, and mechanical behavior of the given biopolymer. The introduction of plastifiers of different chemical structures as modifiers of mechanical and thermal properties has a long history of research and today remains one of the most effective ways to improve the structure and modulate the functional behavior of biopolyethers such as PHB, PLA, as well as their mixtures [21,22,23]. The next category of the modifiers comprises the compounds affecting diffusivity and solubility as well as the permeability of PHB membranes/films designated for separation and purification in liquid media [24] and food packaging materials against atmospheric gases [25]. Such novel barrier materials include layered inorganic fillers such as silicates, whose mechanism of action is determined by the lengthening of the diffusion pathway due to an increase in the tortuosity of the diffusion flow of gas, drug, and solvent molecules [26,27]. In a number of systems, modifying fillers and functional nanoparticles such as iron oxides (Fe_3_O_4_) interact with the ester groups of the PHB, slowing down its segmental mobility and, as a result, reducing the transport characteristics of drugs in polymeric therapeutic systems designed for controlled, targeted release [28].

In recent years, a large number of comprehensive publications devoted to the study of various types of biocomposites with modifying additives, which radically change the performance properties of the original polymeric systems, have been presented in the literature [29,30,31,32]. Within the framework of a brief literature review, we limited ourselves to reviewing the works of the predecessors using graphene and its oxide as two-dimensional (2D) planar nanomodifiers, which significantly improve the characteristics of PHB and its analogs [33,34]. Currently, nanomaterials with 2D topography modify the functional behavior of degradable biocomposites, providing the inventive basis for the design of cutting-edge constructs in biomedical applications, packaging, environmental pollution remediation, electronic devices as well as in other advanced areas of device development and health care activities. Nowadays, 2D nanomaterials find versatile implementation in bio-integrated soft engineering [4] for electronic and bio-sensors [35,36], pharmaceutical delivery platforms [37,38], noninvasive diagnostics, monitoring, and bio-pattern imaging [39,40].

Among the 2D planar materials, graphene (G) and graphene oxides (GOs) are carbon-constituent modifiers with a great specific surface area, about 2.5 × 10^3^ m^2^/g and impressive mechanical behavior expressed in high values of Young’s and elastic modules [41]. Adjusted electrical conductivity, from 10^−17^ to 10^2^ S/m, enables engineers to use graphene derivatives in sensors and miniature electronic devices [42,43,44]. At room temperature, thermal conductivity for G spans 3000–5000 W·m^−1^·K^−1^ and surpasses the analogous characteristic for other carbon-constituent systems such as diamond, carbon nanotubes, graphite, and some others [42]. This special feature of the planar modifiers opens wide horizons for the design of thermosensitive platforms and energy converters [45] and could be implicated in enhancing the thermoelectric performance of polymer composites [46].

The class of GOs comprises the oxidized derivatives of G containing oxygen moieties such as hydroxyl, carboxyl, and epoxy groups. All of the functional groups are located on the edging and main surfaces. The class of GOs comprises the oxidized derivatives of G-containing oxygen groups such as hydroxyl, carboxyl, and epoxy groups. All of the functional groups are located on the edging and main surfaces of the planar entities [47,48]. Nowadays, stoichiometric and molecular structures of GO are still discussed in world publications, and they are essentially varied depending on the synthesis methods [48].

Despite the inhomogeneity of the oxygen groups on the GO surfaces, they are able to interact easily with functional groups of biopolymers, forming a special class of nanobiocomposites with improved or original exploitation characteristics [49,50]. It is worth saying several words on mechanism transport through GO membranes (GOMbs): recently, it was stated that owing to the combination of O-containing and C-containing domains, water permeation across the nanochannels of GOMb proceeds by a partially frictionless mechanism [51,52]. Membrane structure imperfections at nano- and micro-levels generate friction phenomena for water molecule diffusivity [51,53]. So, the ratio of O-containing and C-containing domains on the surface of GOMb is a key factor regulating the ion and water transport rate in nanochannels of GO hybrids composed on the base of various functional polymers [54].

A short time ago, essential progress had been made in the usage of G and GO as therapeutic vehicles for drug targeting in the different areas of medical applications [55]. The next step towards the design of less toxic carriers of drugs was undertaken through the design of GO-biopolymer conjugates that are well known as bionanocomposites where the oxide-containing graphene exhibits not only special functionality but the reinforcing activity to prolong the integrity of therapeutic systems providing the long-term drug release.

In many situations, the biopolymers’ characteristics can be treated with exterior and interior impacts such as temperature, mechanical forces, humidity, UV and γ irradiation, enzymes’ activity, and others. Any of them in a single realization or the multiple impacts combination can negatively affect their exploitation performance. In the comprehensive review, Tayouri, Estaji, Khonakdar, et al. [56] have recently presented the comparative analysis for polymer nanocomposites on the base GO and reduced GO associated with the hydrolytic, enzymatic, photolytic, and thermal degradation of the nanocomposites. Herein, one of the key factors enhancing negative impact resistance is the homogeneous distribution of the GO nanoparticles in a polymer matrix. In our view, it is very important not only for functional optimization of bionanocomposite behavior. In the polymer matrix, the GOs associates could decrease modifier efficacy, but randomly distributed points of interaction of the oxygen groups and biopolymer functional groups (e.g., the ester moieties in polylactides, PHB) should affect macromolecular dynamics controlling such important processes as protein adsorption, water/ion diffusion, drug/gene controlled release, and many others. Given the large number of publications devoted to polymer composites modified by G and GO nanoparticles, it is reasonable to limit their review to the part that concentrates on studies of similar systems, namely, on binary composites of PHB–GO type. At the same time, we will leave aside no less intriguing biodegradable nanocomposites with the participation of initial graphene and other modifying carbonaceous materials, such as pyrolyzed carbon, carbon fibers, nanotubes, and others.

In the first publications related to the synthesis of PHB and graphene nanocomposites, the authors investigated the special structural characteristics reflecting the morphological and crystalline properties of the nanostructured systems, which represent rather an academic than an applied aspect of the development of innovative materials of this type [57,58]. Subsequently, the scientific and instrumental analysis of bionanocomposites has increasingly shifted toward applied developments, especially in the fields of bioengineering, miniaturization, energy converters, and environmentally friendly materials for membrane processes and packaging.

One of the pioneer works devoted to PHB–GO bionanocomposites investigation of the packaging design was presented in [59]. The authors have successfully produced PHB–GO packaging systems and executed following reasonable explorations of mechanical, structural, thermophysical, barrier, and biodegradable features. Compared with the homopolymer PHB, the bionanocomposites displayed enhanced mechanical behavior, a substantially higher melting point (by 10 °C), improved thermal stability, and advanced barrier characteristics relative to oxygen and water. Besides, herein, there is a four-fold increment in the shelf-life of humidity- and oxygen-sensitive food products [59].

Recent investigations have also established that graphene derivatives expose perceptible antibacterial and angiogenic phenomena [60]. The multicomponent curcumin-loaded composite consisting of GO, sodium alginate, and PHB has been elaborated for the treatment of diabetic wounds [38]. These results, in combination with successful clinical trials, show that the bionanocomposite scaffold could be implemented for antidiabetic devices, which should “potentially improve the quality of life for millions of patients” [38]. Much more sophisticated G–PHB nanocomposites with embedded graphene quantum dots have recently demonstrated an ultimately successful recognition of receptor binding domains from the coronavirus spike protein to indicate the specific antibody as the witness of the virus infection [12].

As the structural features of the innovative PHB–GO nanocomposites were examined in depth, improvements in a number of their physical properties were demonstrated [61], the practical use of which will provide new bioengineering platforms for cell and tissue engineering, as well as therapeutic systems for controlled and targeted drug delivery. For example, recent publications by Surmenev et al. [34,62] have shown that the introduction of the reduced GO into the PHB matrix causes an increased piezoelectric effect, the manifestation of which is highly desirable for bone tissue repair, as well as in the functioning of scaffolds and neuroconduits [63,64,65,66].

Taking into account a brief analysis of the previous studies, the present work aims to (a) consider the effect of modification by GO nanoparticles of the structure of ultra-thin PHB fibers obtained by electrospinning, (b) analyze the effect of external factors on the structural and dynamic characteristics of PHB–GO bionanocomposite, and (c) determine the effect of the GO/PHB system component ratio on the structural and dynamic properties of mixture compositions using microprobe ESR and DSC methods [67]. Here, the diameter and cross-sectional geometry of the PHB fibers are also to be evaluated. The study of the processes occurring in bionanocomposites under the influence of temperature, moisture, and/or ultraviolet (UV) is highly advisable in terms of the specific environmental effects, where the aforementioned effects can appear as a single factor or as a complex combination of several aggressive factors [68].

## 2. Materials and Methods

In this work, polymeric nanofiber materials were obtained by the method of electrospinning (ES) on a single-capillary laboratory unit with a capillary diameter of 0.1 mm and the following operating parameters: electric current voltage—12 kV, the distance between the electrodes—18 cm, and the electrical conductivity of the solution—10 μS/cm.

Polymer molding solutions were prepared from a partially crystalline biodegradable polymer poly-3-hydroxybutyrate (PHB) of 16F series (BIOMER^®^, Krailing, Germany). The molecular weight of PHB was Mw = 2.06 × 10^5^ g/mol (206 kDa), density d = 1.248 g/cm^3^. The following solvents were used in obtaining the films by irrigation: for PHB–CHCl_3_ and dioxane of the “ChDA” brand (Ekos1, Staraya Kupavna, Russia). Fibers were obtained by electroforming using a single-capillary laboratory unit with the following parameters: capillary diameter—0.1 mm.

Finely dispersed PHB powder was dissolved in chloroform at 60 °C to obtain molding solutions. The concentration of PHB in the solution was 7 wt. %, the content of graphene oxide was 0.05, 0.1, 0.3 and 1 wt. % relative to the PHB mass. It should be noted that the dopant plates are localized only in the amorphous regions of the polymer, and the share of such regions changes with the increase in the additive concentration. For this reason, the local concentration of the graphene oxide in the amorphous regions of the polymer changes significantly.

Graphene oxide-based additives were used as a modifying agent for the creation of fibrous matrixes. They are as follows: graphene oxide (GO) with the composition C—59.2 wt. %, O—31.9 wt. %, H—8.8 wt. % and partially reduced graphite oxide (RGO) with the composition C—84.0 wt. %, O—10.4 wt. %, H—5.6 wt. %, [23]. RGO was synthesized in the Institute of Chemical Physics, named after N. N. Semenov of the Russian Academy of Sciences, Moscow, Russia. GO was obtained by the authors of this paper following the classical Hammers method [36,37] by oxidation of pencil graphite with sodium nitrate and potassium permanganate in concentrated sulfuric acid. The GO was purified by repeated washing with a 5% hydrochloric acid solution and then with water until sulfate and chloride ions were absent. The obtained in the form of a swollen paste GO suspension was dried in a thin layer at 60 °C. RGO was obtained by “explosive” thermal reduction of GO at 700–750 °C, following a previously described method [38]. The explosive reduction of GO was carried out in a vertical tube furnace on the basis of a quartz tube 50 mm in diameter and 100 cm long, the bottom part of which was hermetically connected with a container for the collection of RGO. The entire volume of the furnace was preliminarily purged with argon. The 50 cm long heating section (temperature 700–750 °C) was located in the lower part of the tube. GO in the form of plates was thrown into the furnace channel. At falling into the hot zone of the furnace, a large amount of gaseous products was released, which resulted in the splitting of graphite oxide particles, a sharp decrease of oxygen content in the material, and obtaining graphene-like material—RGO with a specific surface of 800 m^2^/g and more. The longitudinal dimensions of the plates were 104 × 105 nm, and the thickness was 1.5 nm.

To fabricate PHB-based fibrillar bionanocomposites, graphene oxide (GO) with an elemental composition of C—59.2 wt. %, O—31.9 wt. %, H—8.8 wt. % was used as a modifying agent. GO was obtained following the classical method of Hammers [36,37] by oxidizing pencil graphite with sodium nitrate and potassium permanganate in concentrated sulfuric acid. GO suspension was purified by repeated treatment with a 5% hydrochloric acid solution followed by distilled water until the absence of sulfate and chloride ions. The obtained swollen suspension of the modifier was dried in a thin layer at 60 °C. Further, in order to reduce the brittleness of GO nanoplates, they were partially reduced by the “explosive” thermal method at 700–750 °C, following a previously described method [38]. Thermal reduction of GO was carried out in a vertical tubular furnace, where heating took place in a quartz tube with a diameter of 50 mm and a length of 100 cm, to the bottom of which a container for GO collection was hermetically connected. The entire volume of the furnace was purged with argon beforehand. A 50 cm long heating section was located at the bottom of the tube. GO in the form of plates was rapidly placed into the furnace channel. Upon entering the hot zone of the furnace, a large number of gaseous products were released, the composition of which was not analyzed. The result of such heat treatment was a marked increase in the relative carbon content and a decrease in the percentage weight of oxygen in the plates to the following values: C—84.0 wt. %, O—10.4 wt. %, H—5.6 wt. %. The ratio between the original GO form and its partially reduced rGO form was 53:47 wt. %, respectively. The obtained wafers had the shape of rectangles with sides of average size 10 4 × 10 5 nm and thickness of 1.5 nm. Their total effective specific surface area was ≈ 800 ± 70 m^2^/g.

Electron spin resonance (ESR) spectra of the X-band were recorded on an automated spectrometer ESR-B (Federal Research Center for Chemical Physics, Russian Academy of Sciences, Moscow). The UV power value did not exceed 1 mW to avoid saturation effects. The modulation amplitude was always reliably smaller than the resonance line width and did not exceed 0.5 Gs. The stable nitroxide radical TEMPO was used as a spin probe. The radical was introduced into the fibers from the gas phase at 50 and 75 °C for one hour. The concentration of the radical in the polymer was determined by double integration of ESR spectra; a vacuum-evacuated solution of TEMPO in CCl_4_ with radical concentration ~1 × 10^−3^ mol/L was used as a reference.

The correlation of probe rotation, fixed by the parameter multiplied by τ, was found from the ESR spectra by the formula given earlier [33]:

τ = Δ*H*_+_ × [(I_+_/I_−_)^0.5^ − 1] × 6.65 × 10^−10^ (1)

where Δ*H_+_* is the width of the component of the spectrum in the weak field, and I_+_/I_−_—the ratio of the intensity of the components in the weak and strong field. The error of measurement of τ was ± 5%.

The equilibrium concentration of the adsorbed radical in the samples of the studied compositions of the same mass was calculated using Brucker (Winer). In the process of taking spectra, the amplification was recorded, the sample was weighed, and the radical concentration was calculated in the Origin program.

The samples were examined by the DSC method using the DSC Q-20 device from TA Instruments (New Castle, DE, USA) in a nitrogen atmosphere at a heating rate of 10 deg/min. The average statistical error of thermal effects measurement was ± 3%. Polymer weight was 0.02–0.03 g. Melting enthalpy was calculated using NETZSCH Proteus 4.8.4 software. Thermal analysis was performed according to the standard procedure [39]. Peak separation was performed using the “NETZSCH Peak Separation 2006.01” software.

## 3. Results and Discussions

### 3.1. Influence of Graphene Oxide Concentration on the Morphology and Geometric Characteristics of the Fibrillar Composites

**Figure 1:**
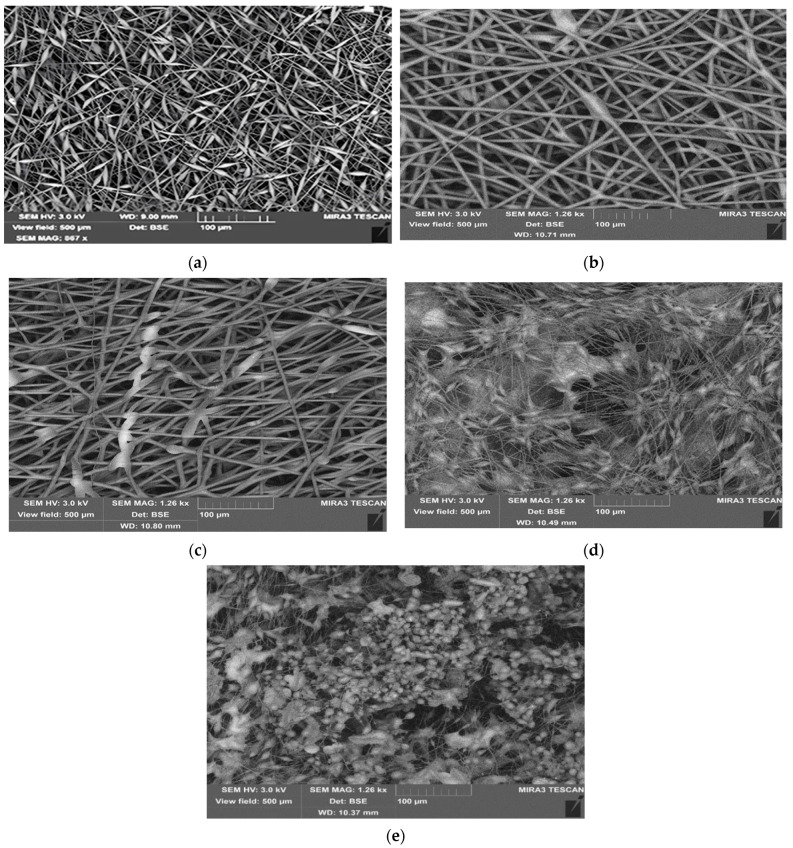
SEM microphotographs of electrospun fibers of PHB loaded with GO (in wt. %): 0 (**a**); 0.05% (**b**); 0.1% (**c**); 0.3% (**d**); 1% (**e**).

Figure 1 shows the microphotographs of the electrospun PHB fibers containing different concentrations of GO. As follows from Figure 1a, during the electroforming process, the PHB fibers form alternating cylindrical and bead-like entities or bead-on-string morphologies [69]. One of the principal reasons for the complex geometry is the poor electrical conductivity of the fiber-formation solution [70]; in our case, the critical electrical conductivity value corresponded to the condition <5 µS/cm. The average diameter of cylindrical fragments belonging to the fiber itself is 4 ± 1 μm, while the diameter of the beads is close to 10 ± 2 μm and their length is 25 ± 5 μm.

With the addition of the electrically conductive modifier, GO, in the volume of polymer solution in the amount of 0.05%, the fiber geometry noticeably improves due to a reduction in the number of thickened beads (see Figure 1b). When the GO concentration is increased by a factor of 2, namely, up to 0.1% (Figure 1c), the bead-like morphology disappears almost completely. In this region of the compositions, the fibers with the plain areas appear, resembling a plane ribbon. The width of the plain sections is 20–30 µm, and their length is several hundred µm. In the structure of the nonwoven material, adhesion arises between adjacent fiber fragments. It is possible that the ribbon-like structures arise due to the interaction of PHB molecules with GO particles.

With further growth of GO concentration in the polymer solution from 0.3 to 1.0%, one can observe the appearance of many spherical anisometric structures and their extended aggregates in the PHB volume. The sizes of discrete sphere-like or ellipse-like structures are 10–30 μm, larger agglomerates are of the order of 50–100 μm, and in some cases exceed 100 μm. The change in the morphology of fiber composites with increasing GO concentration seems to be related to the irregular distribution of nanoplates in the polymer solution due to the tendency of their aggregation in the organic low-polarity solvent. As can be seen in Figure 1d,e, the agglomerates are bound to each other by cylindrical fibrous elements, which can be defined as PHB microfibers. At the maximum GO content (1%), sphere-like morphological elements crucially predominate over the single nanosheets.

For such samples with aggregated GOs, it is quite realistic to assume that the aggregation is immediately initiated in the polymer solution and further developed in the liquid polymer jet during solvent evaporation throughout electrospinning. Due to high adhesion among the nanoplates, their axial orientation in the flow jet practically does not occur, which is confirmed in the microphotographs. The size and shape of agglomerated regions formed by GO nanoplatelets are stochastic. As can be expected, the GO nanoplates affect the regime of electrospinning even at low concentrations and, consequently, modulate the morphology of the biopolymer ultrathin fibers.

### 3.2. Thermal Characterization of the Crystalline Phase of PHB in Its Composites with GO

The mutual influence of crystalline and amorphous regions in biodegradable composites remains a complex issue, and this phenomenon is rather limitedly presented in contemporary publications on polymer science. Meanwhile, the study of the biocomposites’ physical state, including thermal behavior, enables the experts to interpret the impact of a number of external factors such as temperature, ozonolysis, exposure in aqueous medium, UV irradiation on the structural and dynamic characteristics of both the biodegradable fibers themselves (the proper fibrillar filaments) and the fiber material (mats) composed from such filaments.

Herein, in the nanocomposites, the authors have studied the crystallinity of ultrafine PHB fibers as a result of the addition of GO plates with a concentration of 0.05, 0.1, 0.3, and 1%. Since the GO modifier is localized in the amorphous regions of the fibers, and taking into account the crystallinity correction, the local nanoplates’ concentrations in the composite are significantly higher than loaded values, and they are equal to 0.13, 0.28, 0.86, and 2.37 wt. %, respectively. The characteristic feature of thermograms for PHB–GO composites is their asymmetric shape with a low-temperature shoulder, which is caused by the formation of an imperfect crystalline phase in the PHB fibers.

Table 1 collects melting temperatures (Tm) and specific enthalpies of melting (Δ*H*) for all of the PHB–GO composites. It is known from the previous published data that the modifier encapsulation leads to uneven distribution dispersity and high intermolecular interaction between the components of the nanocomposite, which are the vital processes that affect the polymer structure [71,72].

For the PHB electrospun fibers, the addition of the GO modifier plays a dual role, affecting structure–dynamic characteristics oppositely. On the one hand, as a result of the disruption of specific initial interactions among PHB molecules and the increase in the free volume of the composite, in such a way expediting chain segments’ dynamics [73]. On the other hand, the presence of oxygen functional groups in GO, as the reactive entities, provides the interactions of the modifier with the ester groups of PHB, which should lead to segmental mobility reduction. The interplay between the two processes determines the morphological features of the composite, changing the melting or crystallization points and spectral ESR characteristics of TEMPO. The latter effect will be specified below in Section 3.3.

**Figure 2:**
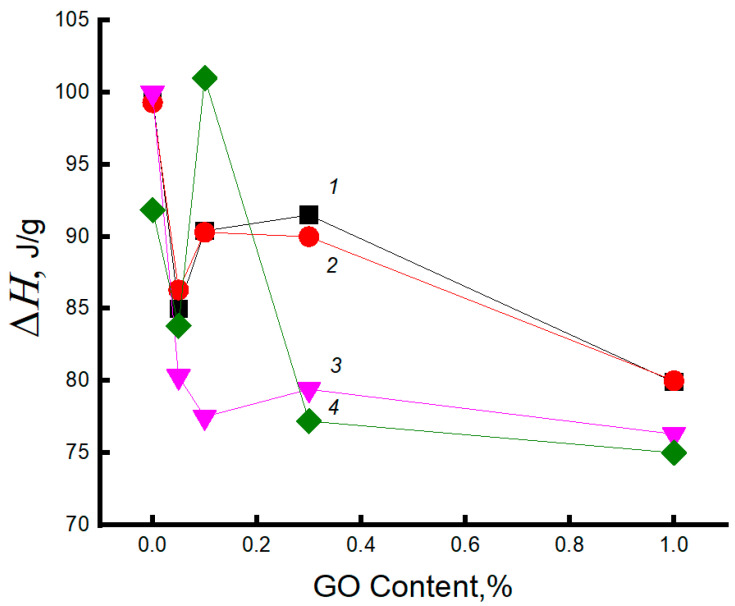
Dependence of melting enthalpy on fiber composition: 1—initial sample, 2, 3—as a result of exposure in aqueous medium at 70 °C for 60 and 240 min, respectively; 4—as a result of ozonolysis for 240 min.

Figure 2 presents data on the changes in the Δ*H* values for the electrospun PHB fibers as the function of GO concentration in the biocomposite. The addition of 0.05% GO leads to a sharp decrease in Δ*H*. At the moderately higher GO concentrations (0.1 and 0.3%), the melting enthalpy increases slightly, and the further growth of the modifier content leads to a decrease in this characteristic. The presence of strong intermolecular interaction of GO with PHB partially prevents the macromolecules from crystallizing, which reflects the decrease in Δ*H* values of the blend with 0.05% GO from 80 to 65 J/g. Therefore, in this region of the GO/PHB ratio, the number of segmental contacts is decreased, the free volume is expanded, and the disordering of the chains prevails over the ordering. Here, a slight increase in Δ*H* is observed in contrast to the previous composition (0.05%), which demonstrates the growth in the ability to pack PHB chains into the crystalline phase as complementary crystallization, with further growth up to 1.0 wt. % GO, the melting enthalpy of PHB decreases monotonically, which is caused by a more intense effect of disordering in the polymer structure, affected by the GO nanoplates.

The fiber geometry presented on the above microphotographs (Figure 1) illustrates that the biocomposite with the 0.1% GO content has the lowest number of defects (thickenings in the form of beds on string) and the fibers with the smallest diameter. It is worth noting that with the increase of GO concentration, the composite melting temperature is decreased from 173 °C for neat PHB to 164 °C for the PHB composite loaded with 1 wt. % of GO that could indicate the formation of a less perfect structure of the crystalline phase.

### 3.3. Segmental Dynamics of PHB–Graphene Oxide Biocomposites

In the previous Section 3.2, the effect of the modifier GO on the crystal structure of the composite was considered. This section is devoted to the analysis of the GO effect on the chain mobility in amorphous areas of the PHB fiber. It is pertinent to note that in highly crystalline polyesters like PHB, the structure of amorphous regions is largely formed under the impact of the crystalline phase, which should be particularly evident in ultrafine fibers. Therefore, GO loading at low concentrations alters fiber morphology, the degree of biopolyester crystallinity, and its chain mobility, especially the amorphous regions. Herein, this dynamic characteristic was investigated by the ESR method using the stable nitroxyl radical TEMPO as the probe being sensitive to segmental dynamics.

**Figure 3:**
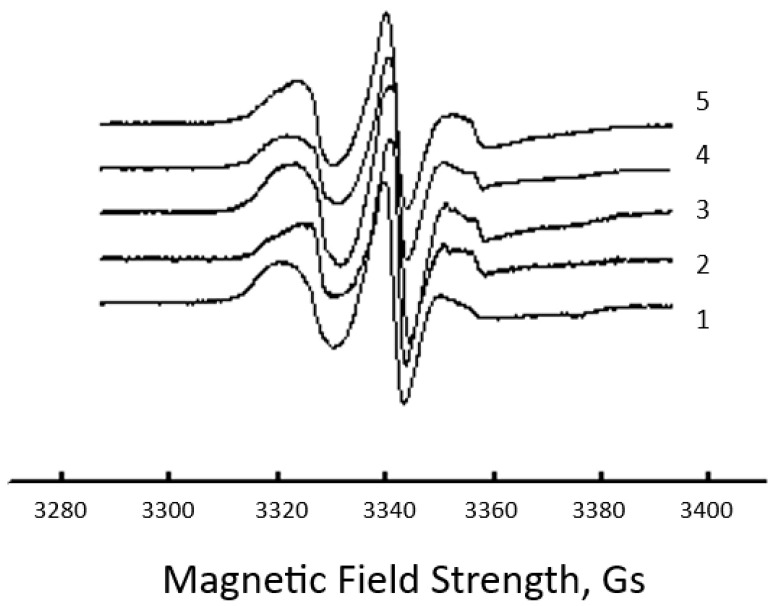
Typical ESR spectra of TEMPO radical in the fibrillar composites of PHB with different concentrations of GO nanoplatelets: 1—0 wt. %; 2—0.01 wt. %; 3—0.1 wt. %; 4—0.3 wt. %; and 5—1.0 wt. %.

It is well known that the structure of the amorphous regions in biocomposites is heterogeneous. It can be represented as a set of structural ensembles with the different conformations and packing densities of the chains in the intercrystalline space and, consequently, with different segmental mobility. Generally, the ESR spectra of the radical in the volume of the neat PHB fibers and its binary blends with a series of synthetic/bio-based polymers have a sophisticated form (like in Figure 3) due to the heterogeneous structure of the amorphous regions and the distribution of the TEMPO probe by rotational mobility. The ESR spectra for the composite under study reflect the superposition of several simpler spectra of radicals located in different regions of the amorphous phase and, therefore, having different mobility.

As a rule, a two-mode structural model of the intercrystalline areas [74,75] with different correlation times τ1 and τ2 is used to evaluate the probe rotation movement, where τ1 characterizes the molecular mobility in denser (rigid) amorphous regions (the slow spectra component) and τ2 reflects mobility in less dense (the soft component) regions (the fast component) as it was shown in Figure 3 and recently [76]. Previously, a computer simulation of the ESR spectra using the Bruker soft program was performed to determine the rotational mobility characteristics of the TEMPO probe [66,67,68]. It should be noted that in the rotational frequency interval of 10^7^–10^8^ s^−1^, the computer simulation of the ESR spectra for the low-molecular-weight probes in polymers is a very sophisticated procedure. In addition, the specificity of the present work is related to the approximate estimation of radical mobility rather than the interpretation of the exact values of the rotational mobility characteristics. Therefore, the authors needed to introduce a simplified mobility feature that could qualitatively characterize the rotation of radicals as the response to chain mobility. It was shown in [77] that the greatest difference in the complex spectra is observed in their high-field and low-field ranges. For this reason, here we chose as an effective characteristic of the spectrum the characteristic rotational correlation time (τ) calculated using the well-known formula [68,78]; see Equation (1) in the Experimental section.

This formula was proposed to calculate the rotational correlation times of the piperidine series nitroxyl radicals in the case of their isotropic rotation in the rotational correlation time region of 5 × 10^−11^ ≤ τ ≤, 1×10^−9^ s. In this region, the ESR spectrum is constituted by three well-resolved components whose widths are described within the framework of Redfield theory [79]. In our case, the τ values qualitatively characterize the shape of the ESR spectrum line and can be regarded as some averaged correlation time.

**Figure 4:**
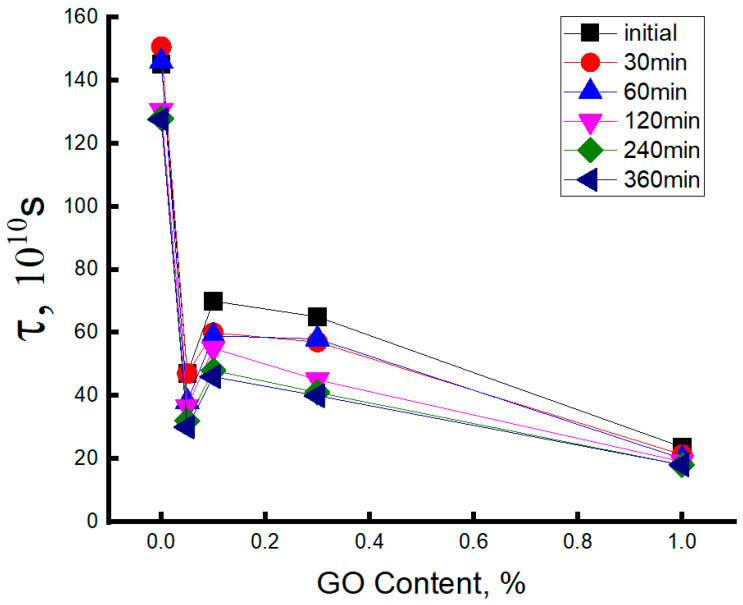
Dependence of correlation time τ on the content of the mixture composition at different times of hydrothermal exposure in aqueous medium at 70 °C.

Figure 4 shows the change in the rotational correlation time (τ) of the radical with the increase in the concentration of the modifier. The character of this dependence has a complex form; namely, with the addition of 0.05% GO, the values of τ have been abruptly decreased (more than three times), further growth of GO up to 0.1% is accompanied by the slowdown in segmental mobility (the moderate increment in τ values) and then, at the higher concentrations of GO, in the interval 0.1–1.0 wt. %, the correlation time is gradually decreased again.

The initial sharp increase in the segmental mobility of PHB is associated with loosening the initial polymer structure under the action of the introduced GO and the growth of the free volume in the system. This effect was described recently for liquid polymer crystals [73]. The subsequent increase in correlation times at a point on the concentration scale corresponding to 0.1 wt % GO is determined by its structuring effect, similar to that presented in the comprehensive works [37,56]. Moreover, the final concentration section in the range of 0.1–1.0%, where the segmental mobility monotonically increases, reflects the tendency for the formation of GO associates (see micrographs in Section 3.1) and, consequently, for the disintegration of the structuring network of nanoparticles.

During the electrospinning process, in the polymer solution/melt jet, the longitudinal, electrodynamic, and hydrodynamic forces affect the macromolecules in the fiber filaments. That exposure is equivalent to drawing the fibrils in the cumulative field of several forces summarizing as an axial tension. If the tension rate is greater than a reciprocal chain retraction time, when the Weissenberg number, Wi > 1 [80], the molecular uniaxial orientation is not dissipated by the viscoelastic flow of the jet and the electrospun polymer has the enhanced trend to flow-induced crystallization [81]. However, the introduced low concentrations of GO nanoplatelets disrupt the ordered structure of the composite fibrils, making them less compact and friable compared with the neat PHB sample. This complex phenomenon is reflected in the rotation behavior of the ESR probe. The corresponding increase in radical mobility, presented as a gradual decrease in the values of apparent correlation time (τ), is presented in Figure 4.

**Figure 5:**
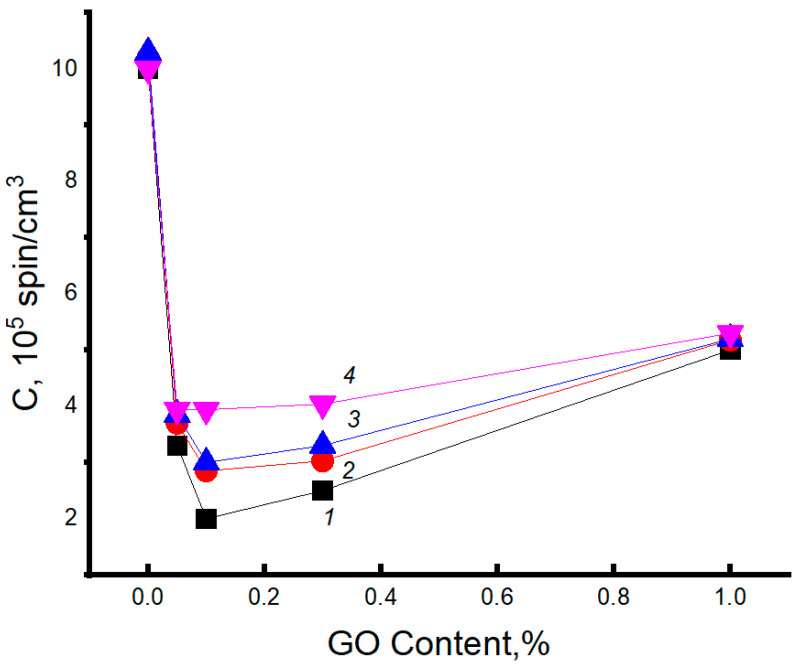
Variation of radical concentration from the composition of bionanocomposite as a result of exposure in hydrothermal conditions for a consecutive period of time: 1—0, 2—60, 3—120, 4—240 min.

Figure 5 shows the equilibrium ESR radical concentration dependencies in fibrillar PHB samples with different GO contents. Basically, these dependencies are anti-symbiotic to the dynamical characteristics of the previous figure. The presence of GO nanoplates impermeable to the radical drastically reduces the permeability of ultrathin filaments with respect to the introduced radical and, hence, its integral accessibility into the fiber volume. However, the subsequent slow growth of the sorption capacity of the TEMPO probe is associated with an increase in the dynamics of polymer molecules, their segmental mobility, and, hence, an increase in the free volume of the fiber.

**Figure 6:**
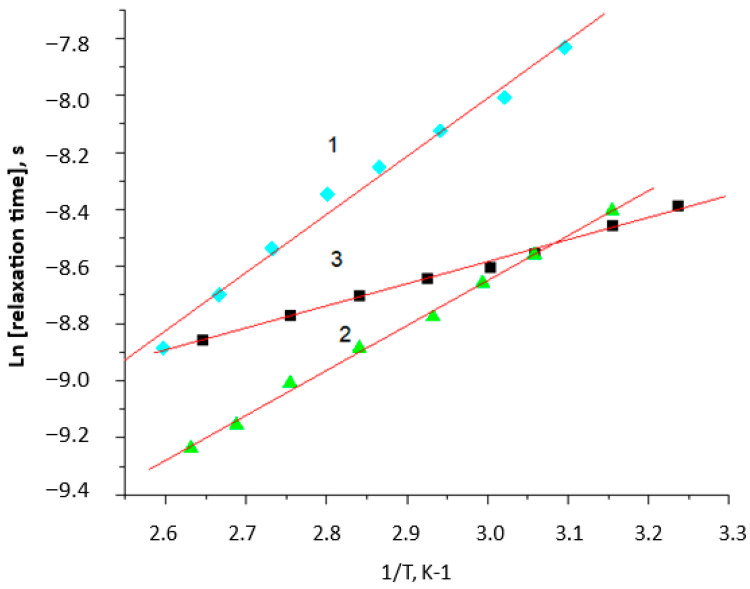
Semilog dependence of time correlation on the reciprocal temperature with different concentrations of the modifier (GO): 1—0, 2—0.1, and 3—1.0 wt. %.

Figure 6 shows the semilog temperature dependences of the apparent correlation time for the neat PHB fibers (a) and the composite PHB–GO (0.1 wt. %) (b). At heating above 30 °C and 60 °C correspondingly, the values of τ are increased with reciprocal temperature linearly. The activation energy of the probe rotation in the PHB composites is equal to 40 (PHB) > 29.7 (PHB–0.1% GO) > 15 (PHB–1% GO) kJ/mol, which indicates the increase in energetical activation barrier for the probe rotation due to looser structural organization in the intercrystalline areas of the composites compared to the neat PHB.

### 3.4. Thermal Characteristics and Segmental Mobility in PHB–GO Composites under the Hydrothermal Effect

Absorption, diffusive mobility, and the state of water molecules in the ultrafine biodegradable PHB–GO fibers are of special interest when considering the hydrolytic and enzymatic reactions or describing the structural evolution in ultrathin fibers under the impact of external aggressive and climatic factors. In this latter case, precipitation and atmospheric humidity determine the service life and decomposition mechanism of the modified biopolyesters, particularly PHB. For example, the structure and molecular dynamics of bio-based polymers greatly influence the diffusion of loaded drugs and, consequently, the kinetic profile of drug release. Since biomedical polymers implanted are regularly subjected to the aqueous environment, it is important to identify changes in the structural and dynamic parameters of bioimplants and medical devices. On the other hand, hydrothermal sterilization of therapeutic platforms and surgical materials could change their structural-dynamic characteristics and, hence, their functional behavior.

In the PHB films and ultrathin fibers, ESR segmental dynamics, equilibrium water sorption, and diffusive transport at physiological and room temperatures have been previously explored in detail in a number of works [82,83,84]. To continue and develop the previous studies and to make the transition from micrometer systems to nanosized ones, it is of interest to evaluate the effect of exposure for the PHB–GO composites in the aqueous medium at the elevated temperature of 70 °C on their thermal characteristic (specific melting enthalpy and melting temperature) and the segmental dynamics. Under this hydrothermal condition, intramolecular hydrogen bonds are largely broken, and the compacted orientation of transitional intercrystalline chains in the fiber is disrupted, which can lead to the partial disintegration of intercrystalline and properly crystalline areas.

A special role in structural transformations of biopolymers belongs to water molecules. Under the hydrothermal impact, its loosening effect is most intensively realized in amorphous regions of fibers, with the growth of segmental mobility that can lead to a more disordered state of intercrystalline regions. As a result of two opposite effects, namely ordering due to plasticizing and disordering due to enhancing molecular mobility, the initial ratio between the less and more ordered regions may be redistributed [85,86,87]. In this section, it is worth discussing a special question—what is the impact of GO nanoplatelets on the complex interplay between disordering and ordering in the nanocomposites?

**Figure 7:**
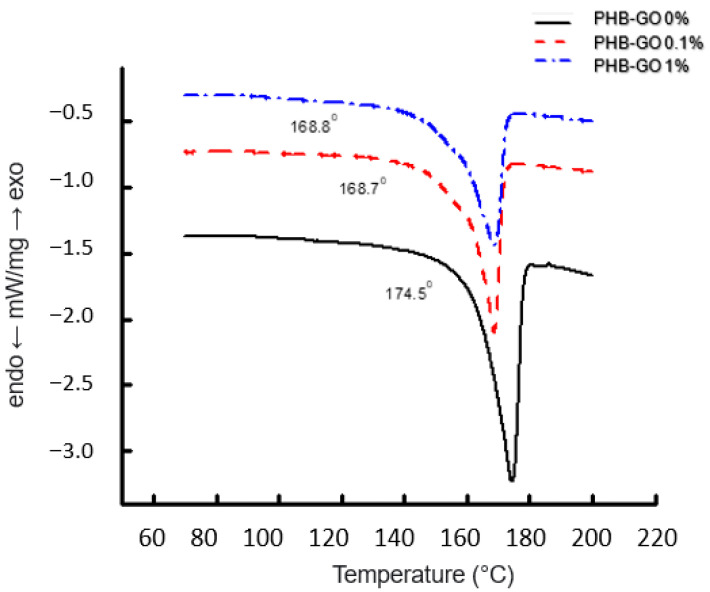
The melting endotherms for PHB fibers with different GO concentrations after the hydrothermal exposition for 4 h.

Figure 7 shows DSC thermograms of fibers for the initial PHB and the PHB–GO composites with the content of the modifying agent equal to 0.1 and 1 wt. %. Combining the results presented in Figure 2 and Figure 7, it is concluded that short-term hydrothermal influence (within 1 h) does not influence the crystallinity of PHB, as it follows from the comparison of dependencies of Δ*H* on GO content (curves 1 and 2). Further exposure of the fibrillar composites for 6 h has resulted in a significant decrease in Δ*H* for all GO compositions: 0.1, 0.3, and 1.0%. The exception is the composition with the lowest GO content (0.05%), where the change in the enthalpy of melting of the PHB crystals is practically absent. Consequently, the stability of the crystalline structure in the biopolymer is limited by the time of hydrothermal exposure, which is probably due to the diffusion of water into the ordered regions of the intercrystalline space. Similar patterns were observed in [85].

A significant decrease in the enthalpy of melting can be explained by the predominance of disordering over the ordering effect. Disturbance in the ordering of the transitional chains is transferred to the end surfaces of PHB crystals with their partial destruction. Earlier, it was shown in [55,56] that chitosan blends with PHB lead to hydrophilization of the binary system.

After evaluating the hydrothermal action, let us consider the effect of exposure duration for the composite fibers on the segmental mobility of the PHB molecules. The simultaneous plasticizing effect of water and increased temperature (70 °C) lead to the intensification of the diffusion mobility of polymer segments and, consequently, to the predominance of the structure disordering effect. In this case, it is natural to observe a decrease in the correlation time, as presented in Figure 3. The increase in the fibers’ exposure duration to the liquid-aggressive medium leads to structural reorganization with increased loosening. The reduction in fiber perfection causes an enhancement in segmental dynamics, which contributes to the fall of τ values.

By analyzing the results of the measurements presented in this figure, it can be easily seen that loading of the minimum concentration of GO in PHB composite (0.05%) results in the most intensive growth of segmental mobility of biopolymer (the sharp minimum of time correlation), which is weakly determined by the time of hydrothermal exposure. The subsequent appearance of the maximum for all curves at 0.1 wt. % indicates a decrease in the molecular dynamics of PHB as a result of the interaction between GO nanoplatelets and ester functional groups. Here, in the region of the second extremum, the compensation of two processes could manifest itself, namely, due to the growth in thermal segmental motion and segmental dynamic inhibition due to PHB–GO interactions. In the interval 0.1–1.0 wt. %, the gradual decrease in probe time correlation (the gradual increase in probe mobility) is the reason for the disruption of the structuring network of GO, owing to the nanoplatelets aggregation and, hence, the growth of PHB molecular mobility.

In the PHB samples subjected to the short time exposure, the probe mobility slows down, while at the long time exposure, this feature is increased. Thus, we can conclude that the short-time contact “composite—water” for several decades of minutes poorly affects the structure in the intercrystalline area, while the long-time exposition for several hours enhances the probe mobility due to an essential disordering of the same intercrystalline area. The exception is the ultimate point on the concentration scale (1.0 wt. %), where the temporal pattern is absent. At this concentration, the aggregation of GO nanoplatelets is maximal and weakly affects the segmental mobility of PHB. Therefore, the probe dynamics are largely free and are not constrained by the nanoplatelet network; hence, the water instantly penetrates the fibrils.

The studies [86,88,89] in which the influence of exposure to aqueous media on the structure of PHB blends with additives have been recently published, where it was shown that, with the increase in the additive concentration, the effect of structure decompaction becomes stronger. For example, in the PHB blends with porphyrin—SnCl_2_ complex (1 wt. %) after 240 min exposition, the probe correlation time decreased by 20 × 10^−10^ s, while in this paper, the values of τ are four times lower, specifically τ = 5 × 10^−10^ s. The essential decrement reflects the polymer structure disordering when the GOs intensively affect the segmental mobility of PHB.

### 3.5. The Impact of Ozonolysis on the Dynamics of Radical Rotation in Fibers of PHB–Graphene Oxide Compositions

In addition to hydrothermal exposure to biomedical and environmentally friendly polymeric materials, medical implants and therapeutic platforms are quite often exposed to the ozone atmosphere. Firstly, this gaseous oxidizing agent is produced by high-powered electrical appliances, both in hospitals and medical institutions and in the high electricity consumption industries. Secondly, the ozonation method is still an effective way to sterilize and disinfect medical devices. Despite the rather widespread applications of ozone with an antibacterial aim, its effect on structural and dynamic characteristics remains poorly investigated. Even fewer publications are known about the interaction of ultrafine fibers with ozone molecules as an aggressive gaseous oxidizer.

It is well known from previous studies that ozone oxidation has a complex effect on the structure and dynamic behavior of the polymer [90,91]. Under the impact of this oxidizing agent, two oppositely directed processes take place, namely, as a result of the appearance of new oxygen-containing groups, the stiffness of the polymer chains increases and, with a certain decrease in their molecular weight, an increase in their flexibility can be observed. The alternative tendencies for a single macromolecule to the uniaxial segmental orientation or to folding into a coil is determined, as noted earlier, by the critical value of the conformational criterion (β*):

β* = h/L = √2/k(2)

where h is the average distance between chain ends, L is the contour chain length, and k is the number of repeated units in the polymer chain. Macromolecules with β > β* tend to have additional orientation in the intercrystalline space, and macromolecules with β < β* tend to move to the conformation of a statistical coil.

In a series of previous works [86,92], the authors have reported that the stressed and elongated polymer chains are oxidized at the highest rate than the non-stressed ones. In the former case, the attack by ozone molecules impacts reactive polymeric centers (the ester functional groups) belonging to (a) the polymer loops that are suspended on the border between amorphous and crystalline phases, (b) the transitional polymer chains packed in the intercrystalline space, and (c) the polymer network formed by hydrogen bonds and covalent bridges at the participation of the new oxygen-contained functional groups having arisen due to ozonolysis. The last two structural entities (b and c) are responsible for the segmental mobility decline and, hence, for the apparent correlation time increment.

**Figure 8:**
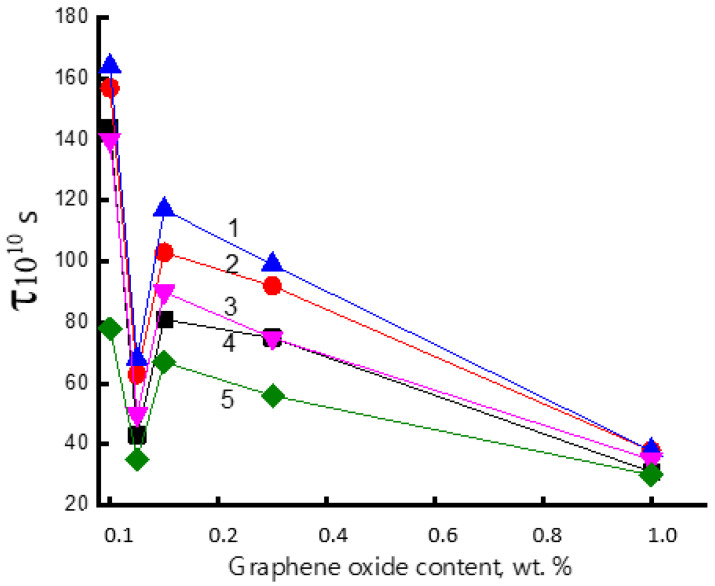
The dependence of apparent time correlation (τ) on the content of PHB-GO composites at the different duration of the hydrothermal exposition: 1—initial composite without ozone exposure, 2—30 min, 3—120 min, 4—240 min, 5—360 min.

**Figure 9:**
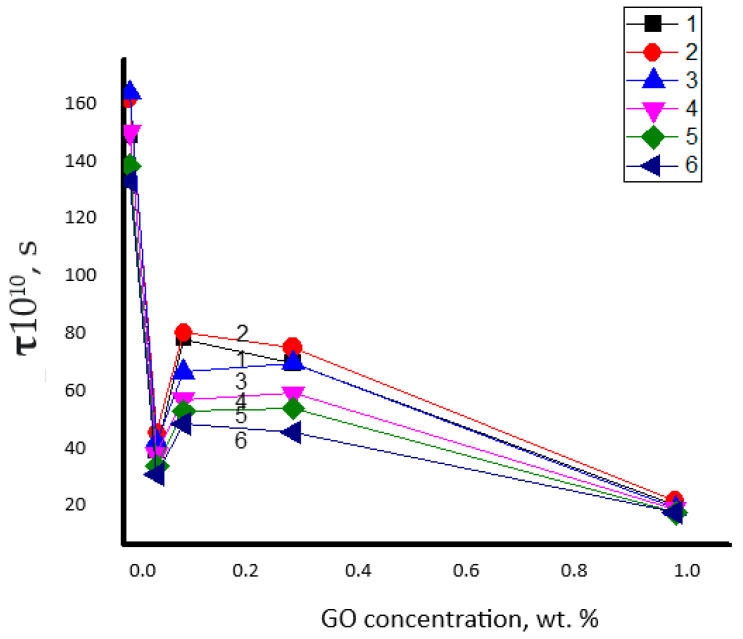
Dependence of the correlation time τ on the composition of the PHB–GO mixture at different durations of ozonolysis. Ozonolysis times: 1—initial composite without ozone exposure, 2—30 min, 3—60 min, 4—90 min, 5—120 min, 6—240 min.

For all exposure times in the ozone atmosphere, in the nanocomposites with a minimum GO content (0.05%), the sharp drop in the correlation times (τ) has already been observed by the first 30 min, is similar to the event observed during hydrothermal exposure (Figure 8). The subsequent gentle maximum and gradual decrease of the dynamic characteristic, τ, have the same reason as in the case of disclosure of composite samples in aqueous medium at an increased temperature (70 °C). Here, too, there is an interplay between the processes of improving chain stacking in the intercrystalline space, which reduces segmental dynamics, and the loosening effect, which acts in the opposite way on the movement of polymer segments. Just as in the previous situation, the time of ozonolysis also affects the macromolecular dynamics. Figure 9 shows the correlation time dependencies of the probe rotational mobility in PHB, which are largely determined by the GO concentration.

Another significant point should be noted: the GO content markedly affects the decrease in τ values. Thus, for example, at maximal loading GO, 1.0 wt. %, the indicated dynamic parameter does not depend on the exposure time, whereas at lower GO/PHB ratios (0.1 and 0.3%), the contact time of the fibers with gaseous O_3_ clearly impacts the segmental mobility of PHB. A preliminary explanation of the decrease in the correlation time presented in Figure 9 comprises the aggregation of GO nanoplates demonstrated in Figure 1. Within this suggestion, at a moderate GO content of 0.1 and 0.3%, the reinforcing network of the nanosized modifier still exists and, to a certain extent, inhibits the ozone diffusion into the PHB fibers. Whereas with the further growth of the GO concentration, the tendency in the aggregation of nanoplates increases, thus reducing the number of PHB–GO contacts and facilitating segmental mobility. Consequently, in this region of the composition (1% GO), the diffusion limitations of the network reinforcing are eliminated, and ozone from the gas phase reaches the reaction centers of PHB almost instantaneously both at 30 and 240 min of exposure, provided that the ozonolysis process passes from the diffusion-kinetic to the kinetic region.

## 4. Conclusions

Research and development in the application of fibrillar bionanocomposites are increasingly attracting the attention of practitioners and scientists. In this work, a study of fibrillar bionanocomposites containing ultrafine GO-modified PHB fibers was carried out, namely: (a) the influence of GO content on the morphology of PHB and the tendency to GO association; (b) conclusions on the influence of external factors, namely hydrothermal exposure, and ozonolysis, on the thermal and segmental dynamic behavior of PHB as the main component of bionanocomposites; (c) evaluation of the effect of GO/PHB ratio on the geometry of electrospun PHB fibers.

The results obtained are interpreted within the framework of two processes oppositely affecting the macromolecular orientation in the intercrystalline regions, where hydrothermal action can act as a factor that thermally destroys the orientation with partial loss of crystallinity, while GO association partially reduces the PHB–GO interaction and, therefore, increases the segmental mobility in the fibers.

The study of structure, morphology, and segmental dynamics in bionanocomposites under the influence of temperature, moisture, and ozone is extremely expedient in terms of assessing the environmental influence on biodegradable fibrillar systems when each of the processes can manifest itself as a separate factor of additive influence on the polymer matrix, or as a complex combination of several aggressive factors, synergistic degradation mechanism, the results of which determine environmental safety from the toxicity of polymeric degradation products.

## Figures and Tables

**Table 1:** Melting enthalpy (Δ*H*, J/g) and melting point of PHB (Tm, °C) of ultrathin fibers of mixtures of PHB with graphene oxide. All characteristics were obtained by DSC.

		Initial			
	PHB	PHB/0.05%	PHB/0.1%	PHB/0.3%	PHB/1%
Δ*H*, J/g	99.7	85	90.5	91	79.9
Tm, °C	172.4	167.6	165.5	167	164
**Ozonolysis for 6 h**					
Δ*H*, J/g	99	84.3	80.7	73.2	73
Tm, °C	175	155; 169	154; 168	155; 169	154; 169
**Exposure to water for 6 h**					
Δ*H*, J/g	99.97	84.3	77.5	84.4	76.35
Tm, °C	174.5	169	168.7	169	168.8

## Data Availability

The data presented in this study are available on request from the corresponding author.

## References

[1] Bosworth L.A., Turner L.-A., Cartmell S.H. (2013). State of the art composites comprising electrospun fibres coupled with hydrogels: A review. Nanomed. Nanotechnol. Biol. Med..

[2] Jayakumar A., Mathew S., Radoor S., Kim J.T., Rhim J.-W., Siengchin S. (2023). Recent advances in two-dimensional nanomaterials: Properties, antimicrobial, and drug delivery application of nanocomposites. Mater. Today Chem..

[3] Ojijo V., Ray S.S. (2013). Processing strategies in bionanocomposites. Prog. Polym. Sci..

[4] Idumah C.I., Nwabanne J.T., Tanjung F.A. (2021). Novel trends in poly (lactic) acid hybrid bionanocomposites. Clean. Mater..

[5] Zafar R., Zia K.M., Tabasum S., Jabeen F., Noreen A., Zuber M. (2016). Polysaccharide based bionanocomposites, properties and applications: A review. Int. J. Biol. Macromol..

[6] Anjum M.N., Malik S.A., Bilal C.H., Rashid U., Nasif M., Zia K.M., Zia K.M., Jabeen F., Anjum M.N., Ikram S. (2020). Polyhydroxyalkanoates-based bionanocomposites. Bionanocomposites Green Synthesis and Applications Micro and Nano Technologies.

[7] Ansari S., Sami N., Yasin D., Ahmad N., Fatma T. (2021). Biomedical applications of environmental friendly poly-hydroxyalkanoates. Int. J. Biol. Macromol..

[8] Pramanik N. (2023). A tool for biomedical application: Synthesis and modification of polyhydroxyalkanoates. Sustain. Chem. Pharm..

[9] Briassoulis D., Tserotas P., Athanasoulia I.-G. (2021). Alternative optimization routes for improving the performance of poly(3-hydroxybutyrate) (PHB) based plastics. J. Clean. Prod..

[10] Di Lorenzo M.L., Raimo M., Cascone E., Martuscelli E. (2001). Poly(3-hydroxybutyrate)-based copolymers and blends: Influence of a second component on crystallization and thermal behavior. J. Macromol. Sci. B.

[11] Bucci D., Tavares L., Sell I. (2005). PHB packaging for the storage of food products. Polym. Test..

[12] Martins G., Gogola J.L., Budni L.H., Papi M.A., Bom M.A., Budel M.L., de Souza E.M., Müller-Santos M., Beirão B.C., Banks C.E. (2022). Novel approach based on GQD-PHB as anchoring platform for the development of SARS-CoV-2 electrochemical immunosensor. Anal. Chim. Acta.

[13] Villegas M., Cid A.G., Briones C.A., Romero A.I., Pistán F.A., Gonzo E.E., Gottifredi J.C., Bermúdez J.M. (2019). Films based on the biopolymer poly(3-hydroxybutyrate) as platforms for the controlled release of dexamethasone. Saudi Pharm. J..

[14] Novikov L.N., Novikova L.N., Mosahebi A., Wiberg M., Terenghi G., Kellerth J.-O. (2002). Novel biodegradable implant for neuronal rescue and regeneration after spinal cord injury. Biomaterials.

[15] Yeo J.C.C., Muiruri J.K., Thitsartarn W., Li Z., He C. (2018). Recent advances in the development of biodegradable PHB-based toughening materials: Approaches, advantages and applications. Mater. Sci. Eng. C.

[16] Garcia-Garcia D., Quiles-Carrillo L., Balart R., Torres-Giner S., Arrieta M.P. (2022). Innovative solutions and challenges to increase the use of Poly(3-hydroxybutyrate) in food packaging and disposables. Eur. Polym. J..

[17] Andrew J.J., Dhakal H. (2022). Sustainable biobased composites for advanced applications: Recent trends and future opportunities—A critical review. Compos. Part C.

[18] Panaitescu D.M., Frone A.N., Nicolae C.-A., Gabor A.R., Miu D.M., Soare M.-G., Vasile B.S., Lupescu I. (2023). Poly(3-hydroxybutyrate) nanocomposites modified with even and odd chain length polyhydroxyalkanoates. Int. J. Biol. Macromol..

[19] D’amico D.A., Manfredi L.B., Cyras V.P. (2012). Crystallization behavior of poly(3-hydroxybutyrate) nanocomposites based on modified clays: Effect of organic modifiers. Thermochim. Acta.

[20] Quispe M.M., Lopez O.V., Boina D.A., Stumbé J.-F., Villar M.A. (2021). Glycerol-based additives of poly(3-hydroxybutyrate) films. Polym. Test..

[21] Khalid M.Y., Arif Z.U. (2022). Novel biopolymer-based sustainable composites for food packaging applications: A narrative review. Food Packag. Shelf Life.

[22] Ray S.S., Banerjee R. (2022). 13—Polylactide—Poly(hydroxyalkanoate) blends. Sustainable Polylactide-Based Blends.

[23] Valente B.F.A., Silvestre A.J.D., Neto C.P., Vilela C., Freire C.S.R. (2022). Improving the Processability and Performance of Micronized Fiber-Reinforced Green Composites through the Use of Biobased Additives. Polymers.

[24] Bandehali S., Sanaeepur H., Amooghin A.E., Shirazian S., Ramakrishna S. (2021). Biodegradable polymers for membrane separation. Sep. Purif. Technol..

[25] Siracusa V., Karpova S., Olkhov A., Zhulkina A., Kosenko R., Iordanskii A. (2020). Gas Transport Phenomena and Polymer Dynamics in PHB/PLA Blend Films as Potential Packaging Materials. Polymers.

[26] Wernert V., Coasne B., Levitz P., Nguyen K.L., Garcia E.J., Denoyel R. (2022). Tortuosity of hierarchical porous materials: Diffusion experiments and random walk simulations. Chem. Eng. Sci..

[27] Khan R.H., Hazra R.S., Nair G., Mohammad J., Jiang L., Reindl K., Jawed M.K., Ganai S., Quadir M. (2022). Cellulose nanofibers as Scaffold-forming materials for thin film drug delivery systems. Int. J. Pharm..

[28] Bychkova A.V., Iordanskii A.L., Kovarski A.L., Sorokina O.N., Kosenko R.Y., Markin V.S., Filatova A.G., Gumargalieva K.Z., Rogovina S.Z., Berlin A.A. (2015). Magnetic and transport properties of magneto-anisotropic nanocomposites for controlled drug delivery. Nanotechnol. Russ..

[29] Shanmugam V., Mensah R.A., Försth M., Sas G., Restás A., Addy C., Xu Q., Jiang L., Neisiany R.E., Singha S. (2021). Circular economy in biocomposite development: State-of-the-art, challenges and emerging trends. Compos. Part C.

[30] Gurunathan T., Mohanty S., Nayak S.K. (2015). A review of the recent developments in biocomposites based on natural fibres and their application perspectives. Compos. Part A Appl. Sci. Manuf..

[31] Shan L., Tan C., Shen X., Ramesh S., Zarei M., Kolahchi R., Hajmohammad M. (2023). The effects of nano-additives on the mechanical, impact, vibration, and buckling/post-buckling properties of composites: A review. J. Mater. Res. Technol..

[32] Kumar P.C.M., Ashok R.B., Kumar M., Roopa C.P. (2022). Natural nano-fillers materials for the Bio-composites: A review. J. Indian Chem. Soc..

[33] Avcu E., Bastan F.E., Guney M., Avcu Y.Y., Rehman M.A.U., Boccaccini A.R. (2022). Biodegradable Polymer Matrix Composites Containing Graphene-Related Materials for Antibacterial Applications: A Critical Review. Acta Biomater..

[34] Chernozem R.V., Romanyuk K.N., Grubova I., Chernozem P.V., Surmeneva M.A., Mukhortova Y.R., Wilhelm M., Ludwig T., Mathur S., Kholkin A.L. (2021). Enhanced piezoresponse and surface electric potential of hybrid biodegradable polyhydroxybutyrate scaffolds functionalized with reduced graphene oxide for tissue engineering. Nano Energy Part B.

[35] Teixeira S.C., Gomes N.O., de Oliveira T.V., Fortes-Da-Silva P., Soares N.d.F.F., Raymundo-Pereira P.A. (2023). Review and Perspectives of sustainable, biodegradable, eco-friendly and flexible electronic devices and (Bio)sensors. Biosens. Bioelectron..

[36] Hashem A.H., Hasanin M., Kamel S., Dacrory S. (2022). A new approach for antimicrobial and antiviral activities of biocompatible nanocomposite based on cellulose, amino acid and graphene oxide. Colloids Surf. B Biointerfaces.

[37] Homem N.C., Miranda C.S., Teixeira M.O., Domingues J.M., Seibert D., Antunes J.C., Amorim M.T.P., Felgueiras H.P. (2022). Graphene oxide-based platforms for wound dressings and drug delivery systems: A 10 year overview. J. Drug Deliv. Sci. Technol..

[38] Chella E.R.A., Kumar N., Nadene N. (2020). Curcumin and Gymnema sylvestre extract loaded graphene oxide-polyhydroxybutyrate-sodium alginate composite for diabetic wound regeneration. React. Funct. Polym..

[39] Mukhortova Y.R., Pryadko A.S., Chernozem R.V., Pariy I.O., Akoulina E.A., Demianova I.V., Zharkova I.I., Ivanov Y.F., Wagner D.V., Bonartsev A.P. (2022). Fabrication and characterization of a magnetic biocomposite of magnetite nanoparticles and reduced graphene oxide for biomedical applications. Nano-Struct. Nano-Objects.

[40] Hosseini E.S., Dervin S., Ganguly P., Dahiya R. (2021). Biodegradable Materials for Sustainable Health Monitoring Devices. ACS Appl. Bio Mater..

[41] Fu X., Lin J., Liang Z., Yao R., Wu W., Fang Z., Zou W., Wu Z., Ning H., Peng J. (2023). Graphene oxide as a promising nanofiller for polymer composite. Surf. Interfaces.

[42] Liu F., Zhang L., Wang L., Zhao B., Wu W., Ray A. (2021). Graphene Oxide for Electronics. Oxide Electronics.

[43] Govindaraj P., Sokolova A., Salim N., Juodkazis S., Fuss F.K., Fox B., Hameed N. (2021). Distribution states of graphene in polymer nanocomposites: A review. Compos. Part B Eng..

[44] Lu X., Munief W.-M., Heib F., Schmitt M., Britz A., Grandthyl S., Müller F., Neurohr J.-U., Jacobs K., Benia H.M. (2018). Front-End-of-Line Integration of Graphene Oxide for Graphene-Based Electrical Platforms. Adv. Mater. Technol..

[45] Wang N., Samani M.K., Li H., Dong L., Zhang Z., Su P., Chen S., Chen J., Huang S., Yuan G. (2018). Tailoring the Thermal and Mechanical Properties of Graphene Film by Structural Engineering. Small.

[46] Xu Y., Li Z., Duan W. (2014). Thermal and Thermoelectric Properties of Graphene. Special Issue: Graphene Research in China. Small.

[47] He H., Klinowski J., Forster M., Lerf A. (1998). A new structural model for graphite oxide. Chem. Phys. Lett..

[48] Mohammed S. (2022). Graphene oxide: A mini-review on the versatility and challenges as a membrane material for solvent-based separation. Chem. Eng. J. Adv..

[49] Ghamkhari A., Abbaspour-Ravasjani S., Talebi M., Hamishehkar H., Hamblin M.R. (2020). Development of a graphene oxide-poly lactide nanocomposite as a Smart Drug Delivery System. Int. J. Biol. Macromol..

[50] Azizi-Lalabadi M., Jafari S.M. (2021). Bio-nanocomposites of graphene with biopolymers; fabrication, properties, and applications. Adv. Colloid Interface Sci..

[51] Deng J., You Y., Bustamante H., Sahajwalla V., Joshi R.K. (2017). Mechanism of water transport in graphene oxide laminates. Chem. Sci..

[52] Moghadam F., Zhai M., Zouaoui T., Li K. (2023). Hybrid graphene oxide membranes with regulated water and ion permeation channels via functional materials. Curr. Opin. Chem. Eng..

[53] Mouhat F., Coudert F.-X., Bocquet M.-L. (2020). Structure and chemistry of graphene oxide in liquid water from first principles. Nat. Commun..

[54] Austria H.F.M., Subrahmanya T.M., Setiawan O., Widakdo J., Chiao Y.-H., Hung W.-S., Wang C.-F., Hu C.-C., Lee K.-R., Lai J.-Y. (2021). A review on the recent advancements in graphene-based membranes and their applications as stimuli-responsive separation materials. J. Mater. Chem. A Mater..

[55] Idumah C.I. (2023). Recent advances on graphene polymeric bionanoarchitectures for biomedicals. JCIS Open.

[56] Tayouri M.I., Estaji S., Mousavi S.R., Khasraghi S.S., Jahanmardi R., Nouranian S., Arjmand M., Khonakdar H.A. (2022). Degradation of polymer nanocomposites filled with graphene oxide and reduced graphene oxide nanoparticles: A review of current status. Polym. Degrad. Stab..

[57] Jing X., Qiu Z. (2012). Effect of Low Thermally Reduced Graphene Loadings on the Crystallization Kinetics and Morphology of Biodegradable Poly(3-hydroxybutyrate). Ind. Eng. Chem. Res..

[58] Beć K.B., Morisawa Y., Kobashi K., Grabska J., Tanabe I., Tanimura E., Sato H., Wójcik M.J., Ozaki Y. (2018). Rydberg transitions as a probe for structural changes and phase transition at polymer surfaces: An ATR-FUV-DUV and quantum chemical study of poly(3-hydroxybutyrate) and its nanocomposite with graphene. Phys. Chem. Chem. Phys..

[59] Manikandan N.A., Pakshirajan K., Pugazhenthi G. (2020). Preparation and characterization of environmentally safe and highly biodegradable microbial polyhydroxybutyrate (PHB) based graphene nanocomposites for potential food packaging applications. Int. J. Biol. Macromol..

[60] Liu S., Zeng T.H., Hofmann M., Burcombe E., Wei J., Jiang R., Kong J., Chen Y. (2011). Antibacterial Activity of Graphite, Graphite Oxide, Graphene Oxide, and Reduced Graphene Oxide: Membrane and Oxidative Stress. ACS Nano.

[61] Cataldi P., Steiner P., Raine T., Lin K., Kocabas C., Young R.J., Bissett M., Kinloch I.A., Papageorgiou D.G. (2020). Multifunctional Biocomposites Based on Polyhydroxyalkanoate and Graphene/Carbon Nanofiber Hybrids for Electrical and Thermal Applications. ACS Appl. Polym. Mater..

[62] Chernozem R.V., Surmeneva M.A., Abalymov A.A., Parakhonskiy B.V., Rigole P., Coenye T., Surmenev R.A., Skirtach A.G. (2021). Piezoelectric hybrid scaffolds mineralized with calcium carbonate for tissue engineering: Analysis of local enzyme and small-molecule drug delivery, cell response and antibacterial performance. Mater. Sci. Eng. C Mater. Biol. Appl..

[63] Pramanik N., Bhattacharya S., Rath T., De J., Adhikary A., Basu R.K., Kundu P.P. (2019). Polyhydroxybutyrate-co-hydroxyvalerate copolymer modified graphite oxide based 3D scaffold for tissue engineering application. Mater. Sci. Eng. C.

[64] Kirkland N.T., Schiller T., Medhekar N., Birbilis N. (2012). Exploring graphene as a corrosion protection barrier. Corros. Sci..

[65] Chen J., Yao B., Li C., Shi G. (2013). An improved Hummers method for eco-friendly synthesis of graphene oxide. Carbon.

[66] Asgharzadeh H., Sedigh M. (2017). Synthesis and mechanical properties of Al matrix composites reinforced with few-layer graphene and graphene oxide. J. Alloy. Comp..

[67] Alam S.N., Sharma N., Kumar L. (2017). Synthesis of Graphene Oxide (GO) by Modified Hummers Method and Its Thermal Reduction to Obtain Reduced Graphene Oxide (rGO). Graphene.

[68] Zhang K., Monteiro M.J., Jia Z. (2016). Stable organic radical polymers: Synthesis and applications. Polym. Chem..

[69] Li T., Ding X., Tian L., Hu J., Yang X., Ramakrishna S. (2017). The control of beads diameter of bead-on-string electrospun nanofibers and the corresponding release behaviors of embedded drugs. Mater. Sci. Eng. C.

[70] Shao Z., Kang G., Chen H., Jiang J., Wang X., Li W., Liu Y., Zheng G. (2023). Preparation, characterization, and air filtration property of electrospun bimodal fibrous membrane based on low conductivity blended polymers solution. Mater. Today Commun..

[71] Shahdan D., Chen R.S., Ahmad S. (2021). Optimization of graphene nanoplatelets dispersion and nano-filler loading in bio-based polymer nanocomposites based on tensile and thermogravimetry analysis. J. Mater. Res. Technol..

[72] Trivedi D.N., Rachchh N.V. (2022). Graphene and its application in thermoplastic polymers as nano-filler—A review. Polymer.

[73] Roilo D., Patil P.N., Brusa R.S., Miotello A., Aghion S., Ferragut R., Checchetto R. (2017). Polymer rigidification in graphene based nanocomposites: Gas barrier effects and free volume reduction. Polymer.

[74] Righetti M.C., Gazzano M., Delpouve N., Saiter A. (2017). Contribution of the rigid amorphous fraction to physical ageing of semi-crystalline PLLA. Polymer.

[75] Klonos P., Terzopoulou Z., Koutsoumpis S., Zidropoulos S., Kripotou S., Papageorgiou G.Z., Bikiaris D.N., Kyritsis A., Pissis P. (2016). Rigid amorphous fraction and segmental dynamics in nanocomposites based on poly(l–lactic acid) and nano-inclusions of 1–3D geometry studied by thermal and dielectric techniques. Eur. Polym. J..

[76] Karpova S.G., Ol’khov A.A., Krivandin A.V., Shatalova O.V., Lobanov A.V., Popov A.A., Iordanskii A.L. (2019). Effect of Zinc–Porphyrin Complex on the Structure and Properties of Poly(3-hydroxybutyrate) Ultrathin Fibers. Polym. Sci. Ser. A.

[77] Chernova D.A., Vorobiev A.K. (2011). Molecular mobility of nitroxide spin probes in glassy polymers: Models of the complex motion of spin probes. J. Appl. Polym. Sci..

[78] Buchachenko A.L., Vasserman A.M. (1973). Stable Radicals.

[79] Shiraishi M., Ikoma T. (2011). Molecular spintronics. Phys. E Low-Dimens. Syst. Nanostruct..

[80] Poole R.J. (2012). The Deborah and Weissenberg numbers. Br. Soc.Rheol. Rheol. Bull..

[81] Nitta K. (2016). On the Orientation-Induced Crystallization of Polymers. Polymers.

[82] Karpova S.G., Ol’khov A.A., Popov A.A., Zhul’kina A.L., Iordanskii A.L. (2019). Analysis of the Structure of Ultrafine Fibers Based on Poly(3-hydroxybutyrate) and Polylactide: Effect of External Factors. Polym. Sci. Ser. A.

[83] Yang H., Sun M., Zhou P. (2009). Investigation of water diffusion in poly(3-hydroxybutyrate-co-3-hydroxyhexanoate) by generalized two-dimensional correlation ATR–FTIR spectroscopy. Polymer.

[84] Iordanskii A., Karpova S., Olkhov A., Borovikov P., Kildeeva N., Liu Y. (2019). Structure-morphology impact upon segmental dynamics and diffusion in the biodegradable ultrafine fibers of polyhydroxybutyrate-polylactide blends. Eur. Polym. J..

[85] Karpova S.G., Olkhov A.A., Popov A.A., Iordanskii A.L., Shilkina N.G. (2021). Characteristics of the Parameters of Superfine Fibers of Poly(3-hydroxybutyrate) Modified with Tetraphenylporphyrin. Inorg. Mater. Appl. Res..

[86] Olkhov A.A., Tyubaeva P.M., Vetcher A.A., Karpova S.G., Kurnosov A.S., Rogovina S.Z., Iordanskii A.L., Berlin A.A. (2021). Aggressive Impacts Affecting the Biodegradable Ultrathin Fibers Based on Poly(3-Hydroxybutyrate), Polylactide and Their Blends: Water Sorption, Hydrolysis and Ozonolysis. Polymers.

[87] Saalmueller J.W., Long H.W., Volkmer T., Wiesner U., Maresch G.G., Spiess H.W. (1996). Characterization of the motion of spin probes and spin labels in amorphous polymers with two-dimensional field-step ELDOR. J. Polym. Sci. Part B Polym. Phys..

[88] Karpova S.G., Ol’khov A.A., Zhul’kina A.L., Popov A.A., Iordanskii A.L. (2021). Nonwoven Materials Based on Electrospun Ultrathin Fibers of Poly(3-hydroxybutyrate) and Complex Tin Chloride–Porphyrin. Polym. Sci. Ser. A.

[89] Tertyshnaya Y., Podzorova M., Moskovskiy M. (2021). Impact of Water and UV Irradiation on Nonwoven Polylactide/Natural Rubber Fiber. Polymers.

[90] Tyubaeva P., Zykova A., Podmasteriev V., Olkhov A., Popov A., Iordanskii A. (2021). The Investigation of the Structure and Properties of Ozone-Sterilized Nonwoven Biopolymer Materials for Medical Applications. Polymers.

[91] Olkhov A.A., Mastalygina E.E., Ovchinnikov V.A., Kurnosov A.S., Popov A.A., Iordanskii A.L. (2023). Biological and Oxidative Degradation of Ultrathin-Fibrous Nonwovens Based on Poly(lactic Acid)/Poly(3-Hydroxybutyrate) Blends. Int. J. Mol. Sci..

[92] Popov A.A., Rapoport N., Zaikov G. (1991). Oxidation of Stressed Polymers.

